# A novel simplified method for extraction of microplastic particles from face scrub and laundry wastewater

**DOI:** 10.1038/s41598-023-41457-y

**Published:** 2023-08-29

**Authors:** C. S. Shalumon, Chavalit Ratanatamskul

**Affiliations:** 1 Department of Environmental Engineering, Chulalongkorn University, Bangkok, Thailand; 2 Center of Excellence in Innovative Waste Treatment and Water Reuse, Faculty of Engineering, Chulalongkorn University, Bangkok, Thailand

**Keywords:** Environmental sciences, Nanoscience and technology

## Abstract

Microplastic pollution in different environmental matrices is a serious concern in the recent times. Personal care products and washing of synthetic fabrics are some of the main sources of microplastic pollution. In this work, a novel simplified, effective and sustainable method for extraction of microplastic particles from face scrub and laundry wastewater was developed. Different parameters affecting the extraction were analysed and the extraction process was optimised. The extraction efficiency of the proposed method was found to be ~ 94.1 ± 1.65%, which was slightly better than the previously available method with an advantage of ease in extraction and lesser time and resource consuming. The developed method was used to demonstrate the extraction of microplastic particles from 12 face scrub samples with different brands. It was found that the samples contained microplastic particles of varying size. The physical and chemical structure intactness of microplastic particles during the extraction was also analysed and found to be acceptable. The developed extraction method was also applied for the extraction of microfibers from the laundry wastewater. It was found that this proposed method is suitable to make the cleaner extracted samples for an easy and more effective qualitative and quantitative analysis of MPs.

## Introduction

The volume of waste materials generated as a result of human activities is increasing in the recent decades. Plastic is the most abundant waste material among those and it contributes about 60–80% of the total waste generated^1^. User friendliness and easy availability of plastic products made their way to the daily life. Production of plastic and similar materials are exceeds 320–335 million tonnes/year^2, 3^. This made the plastic pollution a pressing environment issue all over the globe and the impact is much higher in countries with low recycling rates of plastic products. Plastics which are small, with a size of < 5 mm are defined as microplastics (MPs)^4, 5^. Based on the source of generation, MPs are classified into primary and secondary microplastics^6^. The presence of MPs in different aquatic systems (fresh water rivers and lakes, marine and estuarine areas, etc.) at varying concentration levels has already been reported^7–10^. Also, it was found that MPs can be in various shapes like film, fragment, spherical, fiber and others^11^.

Generally, MPs got long-term stability in different environmental matrices due to their physical and chemical properties^12–14^. This lay the way to exposure with different organisms including human being. Major route of MPs exposure to human body are through inhalation and ingestion. It was reported that the possible ingestion rate can be 39,000–52,000 particles/person/year^15^. Also, the exposure through inhalation is estimated at about 48,000 MPs/day^16^. Thus exposed MPs were reported to be potential toxic to organisms^17, 18^ and may cause oxidative stress and cytotoxicity due to its high surface area, chronic inflammation and cancer due to its persistence in the body^19^. Certain studies reported that MPs may release chemicals from its matrix or adsorbed chemicals from the environment^20^. Also, it may can act as a vector of pathogens which may can cause illness to humans^21^.

Usage of personal care products are one of the primary sources of microplastic generation and washing process of synthetic textiles may contribute to the pollution by means of being a secondary source. A study from China in 2021 estimated that more than 37 billion microbeads was reaching the environment through the wastewater treatment plants^22^. Use of MPs in personal care products were started long back in 1972 when the first patent was granted for the use of plastic synthetic resins as scrubber in skin cleaners^23^. Generally, MPs in personal care products are in the form of “microbeads” which are plastic particles of less than 500 μm in diameter^24^. About 90% of the microbeads used in personal care products are made of polyethylene; but polypropylene and polystyrene are also being used^25^. It was proven that these microbeads from personal care products are rinsed off during usage and end up in drainage systems and then to wastewater treatment systems where it will pass through and find its way to ingestion by animals and human^26^.

Microfiber generation during the washing and processing of fabrics made of synthetic fiber greatly contribute to the microplastic pollution in wastewater^27^. Among different MPs, these microfibers were found to be abundant in water bodies and aquatic organisms^27–29^. It was estimated that about 150,000 fiber particles were got released during the washing process of a kilogram of cloth load^30^. This form of MPs are considered to be imposed the highest threat to the marine biota than MPs in any other shapes^5, 31, 32^. Also, microfiber can act as a carrier of contaminants due to large surface area to volume ratio and high affinity to hydrophobic organic chemicals^33^. Microfibers are most commonly found MPs in the digestive system of aquatic animals^34^ and it cause intestinal blockage and other damages to these organisms^35, 36^.

Many countries like United states, South Korea, Canada, Australia enforced the ban of the usage of MPs in daily usage products earlier. Later, many countries have initiated the same policy and recently China and Argentina also banned the usage of microbeads by the end of 2022. A recent study reported that the non-degradable MPs usage in daily use products are only implemented by a limited number countries and majority of the countries not banned the MPs usage yet^37^. Even if the ban of products in the market was implemented, the usage of the already purchased products by the customers will be continued. Thus, the daily generation of MPs continue and contribute to the pollution. The fact is that this usage and MP pollution during this transition period between the complete ban of microbeads and the complete usage of the already purchased products will be unaccounted. This indicates the need of constant analysis for the MPs occurrence in daily use products even when the legal ban is implemented.

In order to effectively manage the consequences of microplastic on human being and environmental, it is needed to have periodic assessment of microplastic particles present in personal care products and discharged laundry wastewater. Analysis of microplastic includes the collection of samples, extraction of particles and analysing using analytical techniques like FTIR, Raman and similar methods. At present, the methods which are being utilised to extract microplastics involve the usage of different apparatus including heating device, vacuum filtration and membranes. Moreover, these methods are also time consuming. This demands the development of a novel extraction process in which there is simple, less time consuming and less reagents/devices needed. It is interesting to notice that the usage of acetone as an extraction agent for the extraction of MPs from face scrub or related products has not been reported. The thought of making use of the effectiveness of acetone as a solvent to dissolve the face scrub products to separate out the microplastic particles was the driving force for the present work. This research aimed at developing a novel simple and less time consuming, less resource required extraction method for the effective extraction of microplastic particles from commercially available facial scrub using acetone as the extraction agent. The developed method was also used for the extraction of the microfibers from the laundry wastewater. Moreover, this proposed extraction method intended to derive a much cleaner microplastic particle sample by using acetone as an agent for organic matter clean up so that the identification and quantification of the final MP samples with the micro-FTIR analysis will be easier and convenient.

## Experimental methods

### Sample preparation and wastewater collection

A commercially available face scrub was purchased from the local shop and made use as the study material for the development of MP extraction method. This product was intentionally chosen from the left over of old stock since the products which are being marketed in Thailand in recent times are free of MP due to the revised norms. This sample was named as “FS1”. Laundry wastewater samples were collected from the student’s dormitory of Chulalongkorn University, Bangkok and made use for studies on the microfiber extraction. Briefly, a volume of five litres of laundry wastewater was collected three times during the operational hours in a clean glass vessel and mixed together to form a representative sample. The samples are stored in 4 °C before usage.

### Extraction method development

**Figure 1:**
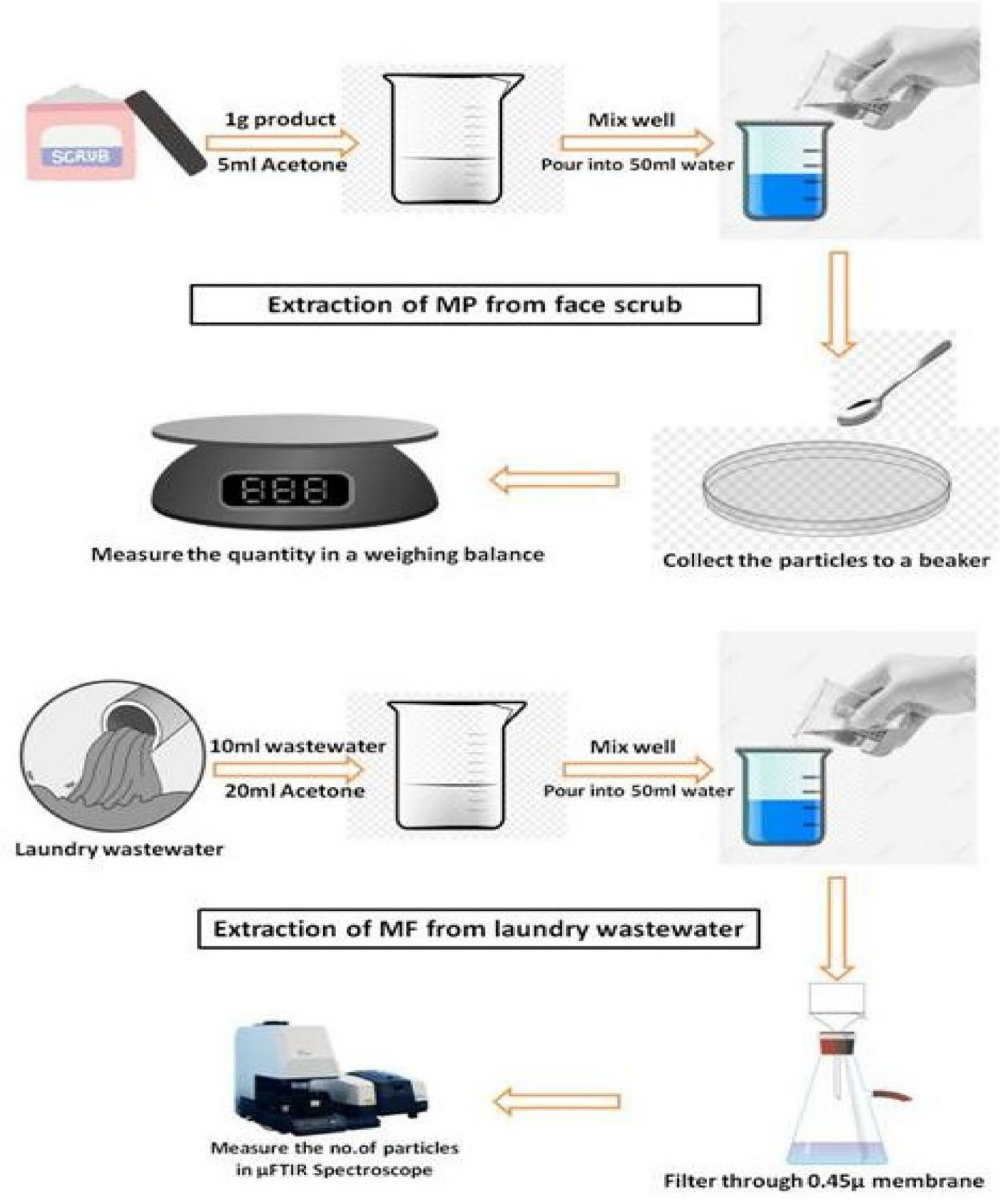
Schematic diagram of the proposed novel method of MPs extraction from face scrub and laundry wastewater.

An extraction protocol was developed to separate and quantify the MP in face scrub and laundry wastewater. Acetone was used in the extraction process and a schematic representation of the protocol is illustrated in Fig. 1. In brief, 1 g of the facial scrub sample (FS1) was taken in a clean glass beaker and 5 mL of acetone was added. The resultant mixture was shaken and poured into another glass beaker containing 50 mL of deionised water. Microplastic particles floating on the water surface was collected and transferred to a glass petri dish using a stainless-steel spoon. Thus, the collected particles were allowed to dry overnight in room temperature and quantified by estimate the weight using a standard weighing balance. Effect of different factors (sample to acetone ratio, acetone concentration and extraction time) on the efficiency of the extraction protocol was also studied by spiking pre-extracted polyethylene MPs (average size of 242 ± 74.9 µm) at a concentration of 20 mg/g of the face scrub sample. A blank sample without the addition of MPs was included. Efficiency of the method (Recovery %) was calculated by dividing the extracted amount of MPs (in mg) by the spiked amount of MPs (20 mg). Each experiment was conducted in duplicates and the results were statistically presented as mean ± standard deviation.

Laundry wastewater sample is thoroughly mixed to ensure the even distribution of the microfibers. A 10 mL-laundry wastewater was immediately measured out into a clean glass beaker. The usage of this volume of wastewater was used to intentionally avoid the crowding of microfibers on the glass filter surface. A volume of 20 mL of acetone was added it the beaker and mixed well. The resultant mixture was poured into a glass beaker containing 50 mL of deionised water. The solution was further filtered through a 0.2 µm glass fiber filter using a vacuum filter device. The membranes were stored in a glass petri dish until analysis.

### Characterization and quantification of MP using micro-Fourier transform infrared spectroscopy (micro-FTIR)

The size and polymer type of the extracted particles were analysed using micro-FTIR in attenuated total reflectance mode (ATR) with the help of a micro-FTIR spectroscope from Bruker, Germany (Bruker-Lumos II). The extracted particles were analysed by the micro-FTIR using Focal plane array (FPA) detector in ATR mode. Spectra were recorded in the wavelength range of 4000–400 cm^−1^ and an average spectrum of 32 scans was derived by using the Bruker OPUS 7.2 software. The polymer type was identified by comparing the spectra with the standard database library of Bruker. The size of extracted particles was determined by using the dimension measuring tool in OPUS 7.2 software.

### Physical and chemical structural aberration analysis

X-ray diffractometer (XRD, Bruker D8 Advance, Germany) with Cu Kα radiation (λ = 0.1542 nm) in the 2θ angle range between 5° and 80° using 0.02° min^−1^ of scan rate was used to characterize the crystal structure of MPs extracted from the sample FS1 using the newly proposed method and the previous method. The characteristic XRD peaks of polyethylene particles were compared to elucidate if there is any change in crystalline structure of the particles due the extraction using acetone. Chemical functionality of the polyethylene particles was compared using the FTIR spectra derived in ATR mode (Bruker-Lumos II, Germany). Zeta potential of the polyethylene particles was analysed using Dynamic Light Scattering (DLS) analyzer (Microtrac MRB Nanotrac wave II, Germany) and compared to elucidate any possible changes in the surface charge of the particles. Scanning Electron Microscopy (SEM) was used to analyse the surface structure of the MPs extracted from the sample FS1. High resolution microscopic images of the particles extracted in the traditional method and the newly derived method were captured using a scanning electron microscope (Quanta FEG 250, Czech Republic). Images of particles extracted using two different methods were compared to figure out there is any differences in the surface structure of the particles which may possibly can happen due to the usage of acetone for the extraction.

### Validation of the extraction protocol

Extraction efficiency of the developed extraction protocol was validated by comparing with a standard method available in the literature. For this, a known amount (20 mg) of the polyethylene MPs were added to the face scrub sample. The extraction was conducted using the method reported by Enfrin et al.^38^ and the newly developed extraction protocol from this research to elucidate the recovery percentages for the efficiency comparison. Pre-optimised extraction protocol was further validated by applying the method with other 12 samples of face scrubs produced by other brands. These products were collected from the customers and collected based on the assumption that these products were produced before the MPs ban in Thailand and still being used by the public. Collected samples were extracted using the new developed method and quantified. Experiments were conducted in duplicates and the statistical analysis in terms of mean ± standard deviation was reported. Microfiber extraction and analysis from laundry wastewater was also compared with the tradition membrane filtration method described elsewhere^39^.

### Quality control/quality analysis

The present study was done under a set of precautionary measures to keep the quality control. All the sample collection and processing were done by using clean glass vessels. Usage of any kind of plastics vessels or tools was avoided intentionally to avoid the contamination. The vessels and tools were washed and rinsed with ultrapure water before usage. Cotton clothes and lab coat were used during the experiments. The processed samples were stored in glass petri dishes and covered with aluminium wrap to avoid the possible contamination. Blank samples were run during the analysis and the experimental values were subtracted from the values obtained for the blank sample.

### Statistical analysis

All the experiments were conducted in duplicates and the mean and standard deviation were calculated. The results were expressed as mean ± standard deviation.

## Result and discussion

### Effectiveness of the new method

**Figure 2:**
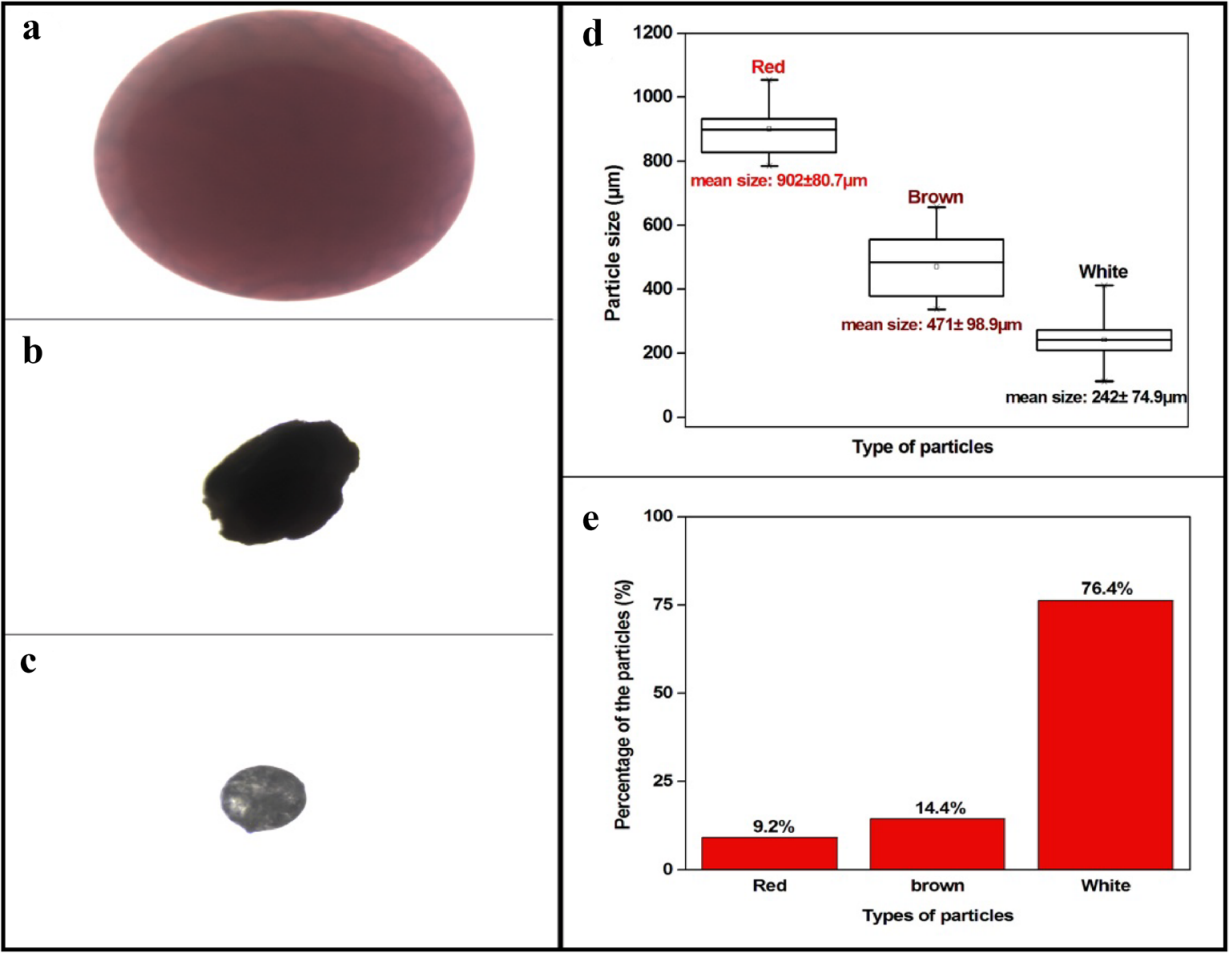
Optical images of different MP extracted from face scrub: (**a**) red (**b**) brown (**c**) white (**d**) particle size distribution of extracted particles (**e**) percentage composition of different MPs in the face scrub sample FS1.

Samples of a commercially available face scrub (FS1) were used to extract the microplastics using acetone as an extraction agent. It was found that the face scrub dissolved well in acetone and leaves out the non-soluble part and MPs. Other contents of the face scrub were got dissolved or precipitated upon addition of acetone. From Fig. 2, the optical images show that MPs were released from the composition of the face scrub, and they got separated. Smaller sized MPs, especially white coloured MPs (average size of 242 ± 74.9 µm) easily got separated and then floated on the surface. This proposed method can make the whole extraction process simple and easier than other methods.

The selected commercially available face scrub was found to contain 3 types of particles with different colours and size as red, brown and white particles (Fig. 2a–c). Red MPs had an average size of 902 ± 80.7 µm, whereas the average size of brown MPs was 471 ± 98.9 µm and the average size of white MPs was 242 ± 74.9 µm as illustrated in Fig. 2d.

The micro-FTIR analysis found that all the MPs were polyethylene particles. It has been reported that polyethylene is one of the most used MPs in the face scrub^40–42^. The reason for wide usage of polyethylene beads in face scrub is due to their surface smoothness which causes less damage to the skin and the resultant redness^43^. Size distribution of each different type of MPs is expressed in Fig. 2c. It was found that the percentage composition of each type of MPs was different in the sample FS1. Red MPs were of 9.2%, whereas brown and white MPs were accounted for 14.4% and 76.4% in the composition as given in Fig. 2e. This reveals that the smaller sized MPs are more in the composition and possibly be a serious environmental burden after the usage of the product.

### Factor affecting the extraction of MP from face scrub

**Figure 3:**
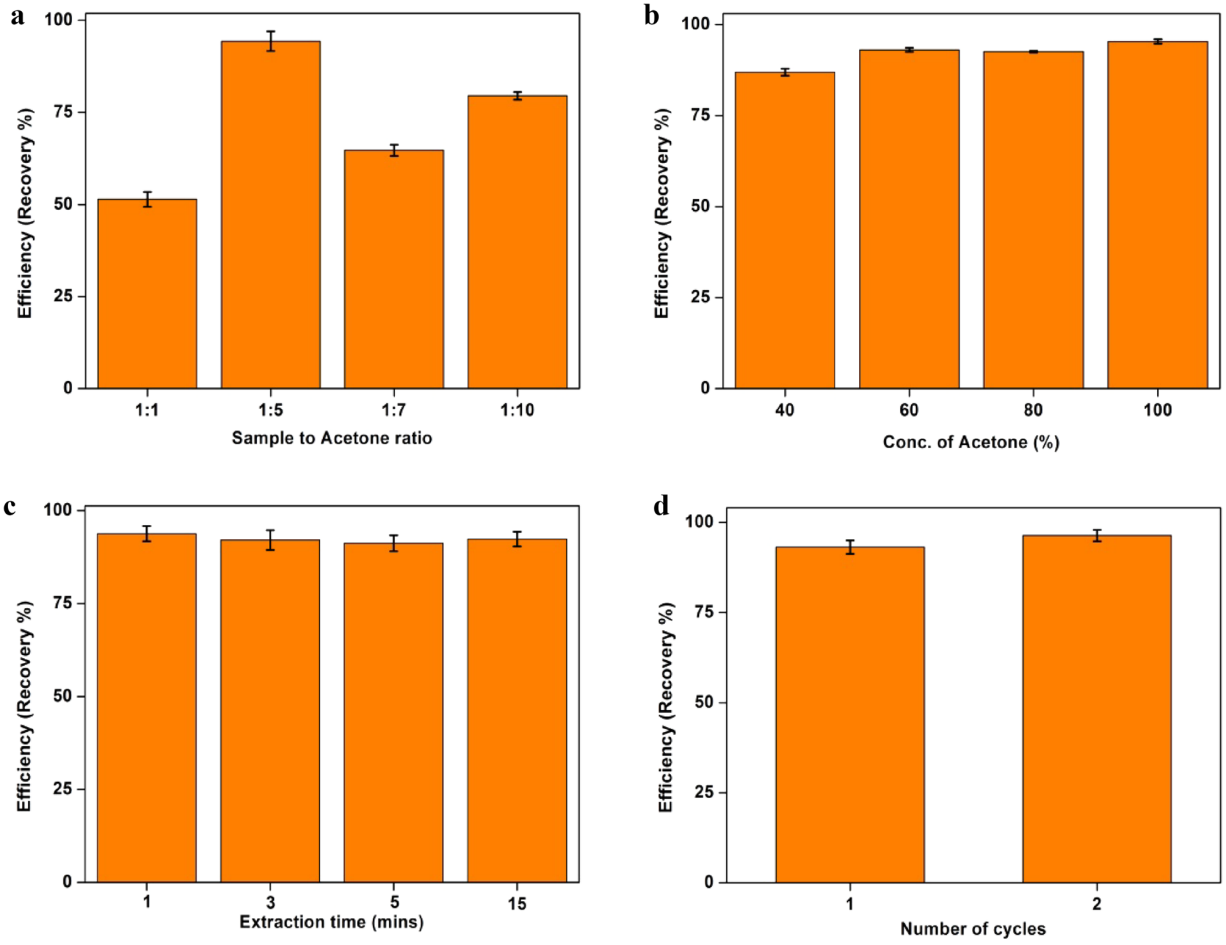
MP Extraction efficiency of the extraction process as a function of (**a**) sample to acetone ratio (**b**) concentration of acetone (**c**) extraction time and (**d**) number of cycles.

Effect of different parameters on the extraction efficiency of the extraction protocol was studied. The efficiency was elucidated in terms of the recovery % of spiked MPs in 1 g of the face scrub sample FS1 (Fig. 3). All the experiments were conducted in duplicates and the results are statistically expressed as mean ± standard deviation.

#### Effect of sample to acetone ratio

Effect of sample to acetone ratio was analysed by varying the volume of acetone from 1 to 10 ml (1:1, 1:5, 1:7 and 1:10) to extract 1 g of face scrub. It was found that the acetone volume has considerable effect of the extraction efficiency of the method (Fig. 3a). The highest recovery % (94.3 ± 2.6) was found at 1:5 ratio and the lowest (51.39 ± 2%) was found for the 1:1 ratio. The least extraction efficiency obtained in 1:1 ratio is possibly due to the insufficient volume of acetone to completely dissolve the contents of face scrub.

#### Effect of acetone concentration

Effect of varying concentrations of acetone as 40, 60, 80 and 100% on extraction efficiency was studied and the results are given in Fig. 3b. It was found that a concentration of 60% and above have the maximum productivity and 40% acetone had lesser productivity in terms of the amount of MP extracted. This is most likely due to the inadequate concentration of acetone in the extraction solution which may possibly lead to the incomplete dissolution of the face scrub product. This in turn may reduce the release of MPs from the bulk of face scrub product.

#### Effect of extraction time

A study on the effect of extraction time on the extraction efficiency was also studied by varying acetone exposure from 1 to 15 min. As evident from Fig. 3c, there was no significant difference in the extraction efficiency at varying acetone exposure time. This shows that the minimum time applied (1 min) is sufficient to separate MPs from the other components of the face scrub. This makes the extraction process much easier and faster than other methods.

#### Effect of number of cycles

The effect of repeated extraction steps on for the complete extraction of MP was studied by comparing one and two cycles of acetone addition and extraction (Fig. 3d). It is evident from the Fig. 3d that there is no considerable difference in extraction efficiency of single and double cycle of extraction process. This indicates that a single step of acetone addition and exposure is enough to completely separate the maximum MP from face scrub. This also gives advantage of less time and chemical consumption for the complete extraction of MPs in a short and simple step. Thus, a concentration of acetone above 60% at a sample to acetone ratio of 1:5 was found to be effective for the successful extraction of MPs from sample FS1.

#### Comparison with previous analysis method

**Figure 4:**
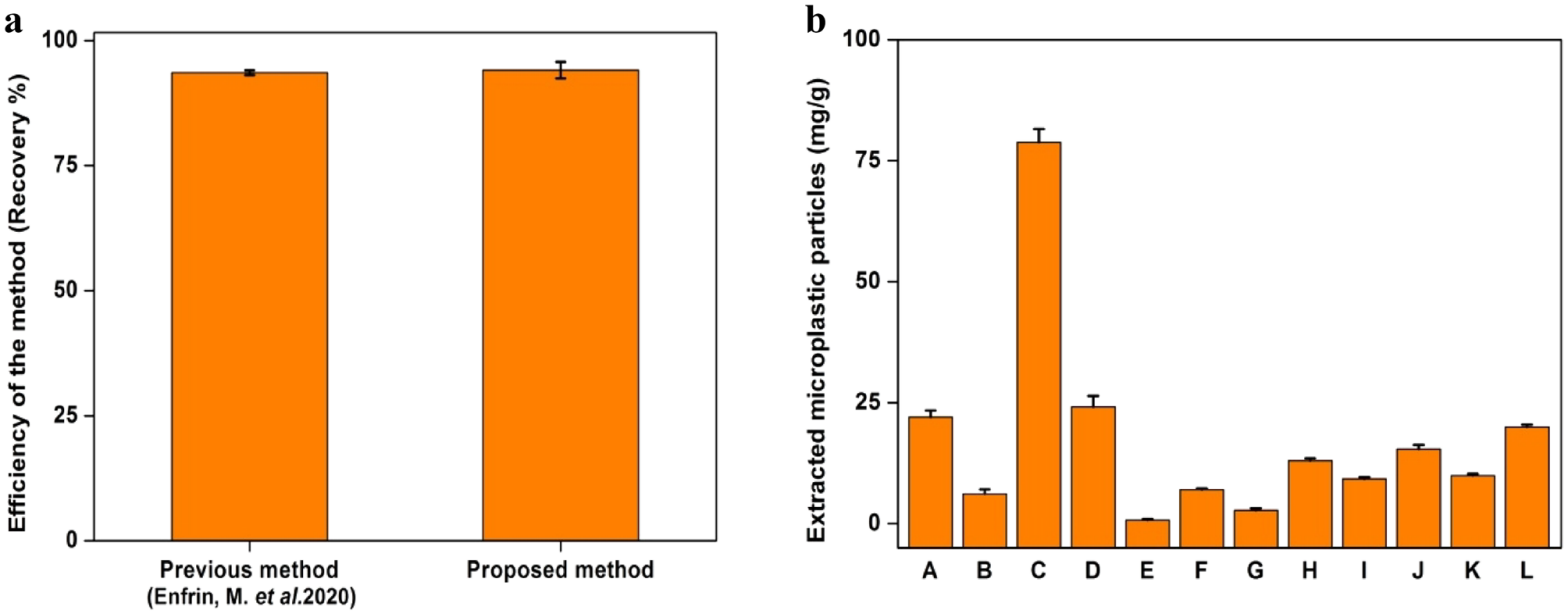
(**a**) Efficiency comparison of the previously available method and the method reported in this study as a function of recovery % of a known amount of MPs in sample FS1 (**b**) concentration of MPs in samples of other selected brands of face scrub.

Newly developed method in this study was investigated and compared with the previous method of MPs extraction by Enfrin et al.^38^ for the microplastic particle extraction from the face scrub by estimating the extraction efficiency from 1 g of the face scrub spiked with 20 mg of MPs, through each method and the results are given in Fig. 4a.

About 93.6 ± 0.5% extraction efficiency was observed for the previous method^36^, whereas the new proposed method of extraction with acetone was able to extract ~ 94.1 ± 1.65% of MPs. This shows that the newly developed method is efficient enough to perform the extraction more or less equal to the previous method. On the other hand, the new proposed method need very less time (1 min) for the extraction and does not require any heating process or membrane devices.

#### MP extraction from other brands

12 face scrub samples belonging to different brands were collected from the users to be used in the validation of new extraction protocol. 1 g of each of the products was subjected for the extraction process and the resultant yield of MPs was expressed in Fig. 4b. It was found that the quantity and colour of the MPs were varying in each product. The presence of MPs varied from 0.75 ± 0.25 to 78.8 ± 2.7 mg/g. The micro-FTIR analysis of the extracted MPs revealed that majority of these particles was polyethylene particles. During the extraction process, it was observed that the face scrub product was well dissolved in acetone and left out the MPs which made the extraction of MPs easier. Thus, it can be concluded that the method could be useful to extract MPs from similar products from different brands. A comparison on the presence of MPs in personal care products established in other studies were compared with the present study and tabulated as Table 1.

**Table 1:** MPs identified from different personal care products.

Source	MP type (size)	Abundance	References
Shower gel	Polyethylene (422 ± 185 μm)	17.80 ± 7.50 mg	^44^
Toothpaste	Polypropylene (3–145 μm)	19,543–52,342 MPs/g	^45^
	Polyethylene (4–20 μm)	0.25–4.17 g particles/10 g	^46^
	Cellophane, polypropylene, polyvinyl chloride, polyamide (3.5–> 400 μm)	327–832 MPs/g	^47^
Facial cleansers	Polyethylene (313 ± 130 μm)	25.04 ± 10.69 mg/g	^48^
	Low density polyethylene and polypropylene(10–178 μm)	11,776–36,636 MPs/g	^45^
	Polyethylene (313 ± 130 μm)	0.75 ± 0.25–78.8 ± 2.7 mg/g	Present study

### Physical and chemical structural aberration analysis

**Figure 5:**
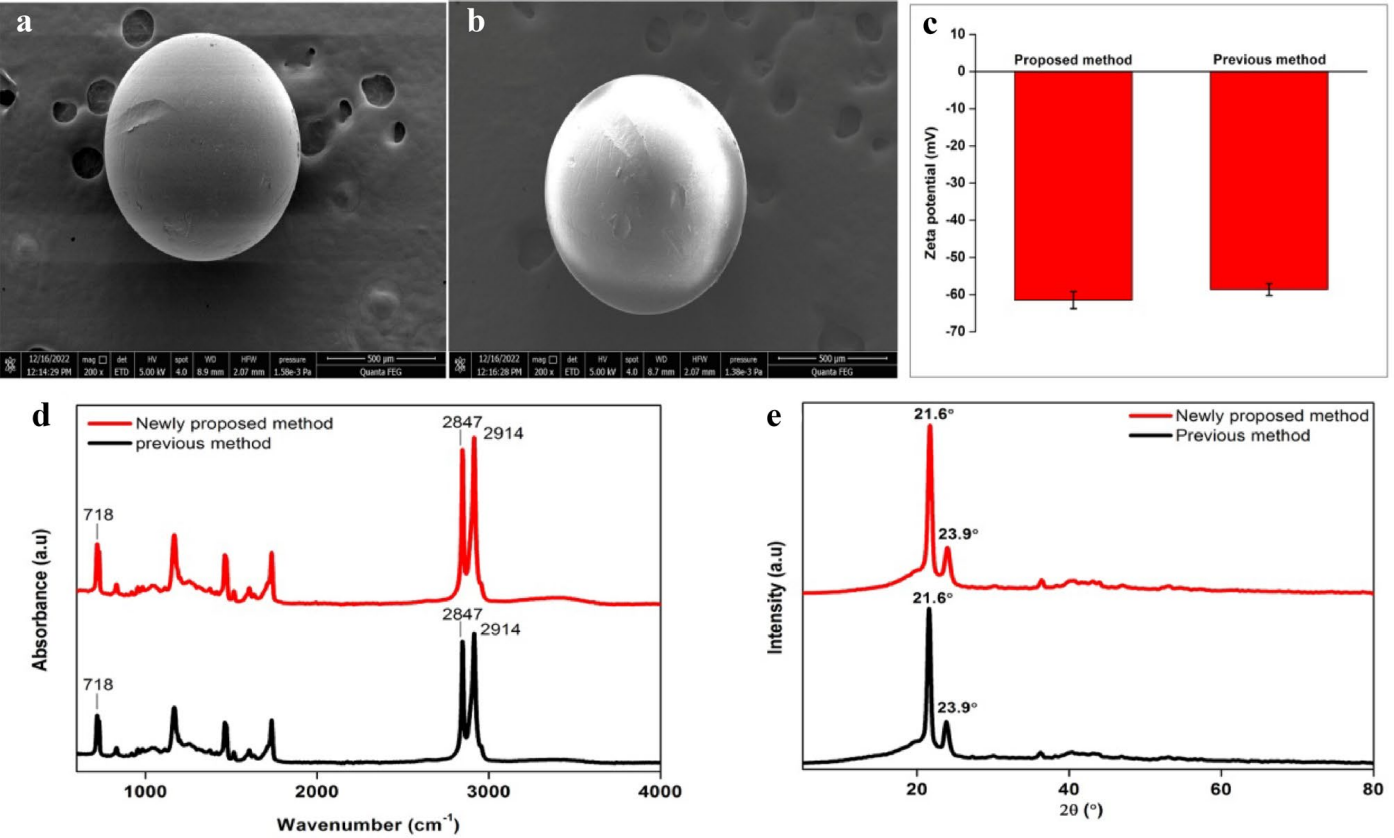
Scanning electron microscopic images of MPs extracted by the newly proposed method (**a**) and the previous method (**b**) comparison of zeta potential (**c**) FTIR spectra (**d**) X-ray diffractogram (**e**) of MPs derived in newly method and previous method.

The chemical functionality, crystallinity, zeta potential and morphology of the Polyethylene MPs extracted in previous method and newly proposed method were analysed and compared to see any physical or chemical aberration were happened during the extraction process with acetone (Fig. 5). High resolution images of the particles extracted from sample FS1 using two different methods were captured and analysed to study the surface stability and intactness of the particles during the extraction using acetone.

SEM images (Fig. 5a,b) of the particles extracted from the face scrub using the previously available method and the newly developed method were compared to see any deformities on the spherical structure of the particles. As evident from the Fig. 5a,b, there is no considerable variation in the spherical structure of the particles extracted by using the previously available method and the newly developed method. This shows that the extraction using acetone doesn’t make any physical structure aberrations to the MPs and the surface is intact. Surface charge of the MPs collected through two different methods of extraction were analysed by measuring the zeta potential of the MPs (Fig. 5c). It was found that there is a negligible difference (less than 5%) in the zeta potential of the MPs extracted between the two different methods. This shows that the surface charge characteristics of the MPs were intact after the extraction with acetone and not much change by the extraction process. To study the chemical functionality of the MPs, micro-FTIR analysis was employed (Fig. 5d).

Characteristic infrared absorbance peaks of Polyethylene at 2914 cm^−1^, 2847 cm^−1^ and 718 cm^−1^, which corresponding to the CH_2_ asymmetric C–H stretching, CH_2_ symmetric C–H stretching and C–CH_2_ bending vibrations, respectively^49^, were observed in FTIR spectra of the particles extracted using previously reported and newly proposed methods. The presence of these characteristic peaks in both spectra shows that the chemical functionality is being preserved and no chemical functionality changes were happened even during the extraction using acetone as an extraction agent. The crystallinity of the MPs was analysed using the XRD spectroscopy (Fig. 5e). It was found that the MPs extracted through both methods contain similar characteristic peaks of polyethylene in diffractogram. In Fig. 5c, the peaks at 2θ = 21.6° and 2θ = 23.9° are the characteristic diffraction peaks of the polyethylene^50^. The sharpness of these characteristic peaks denotes that the particles possess crystalline structure. Thus, it is evident that the extraction process with acetone didn’t make any changes to the crystalline structure of the MPs during the extraction process. In general, it could be concluded that chemical functionality, crystallinity, zeta potential and morphology of the Polyethylene MPs were also being preserved during the extraction process with acetone. This indicates that the proposed method is safe to use to extract the MPs without making any changes in its properties.

### Applicability of extraction method for microfiber extraction from laundry wastewater

**Figure 6:**
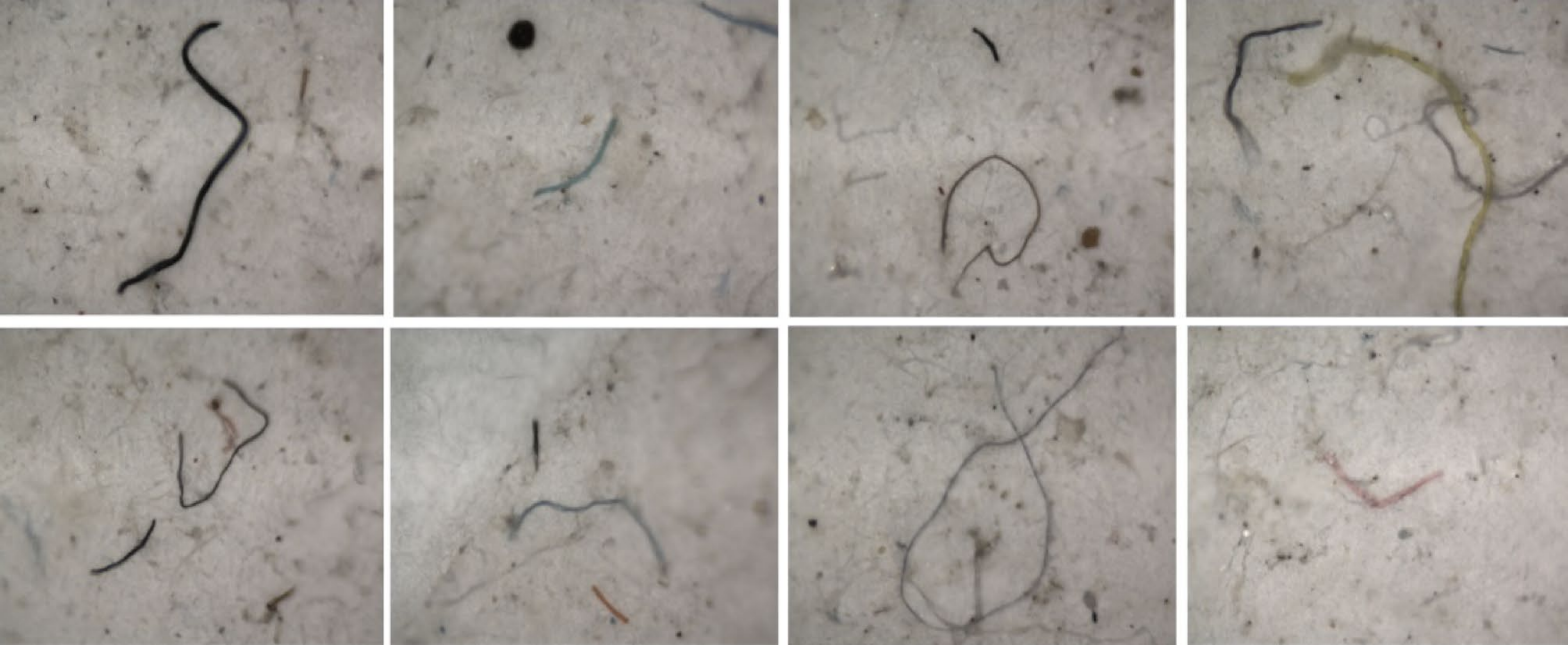
Optical images of different microfibers extracted from the laundry wastewater.

**Figure 7:**
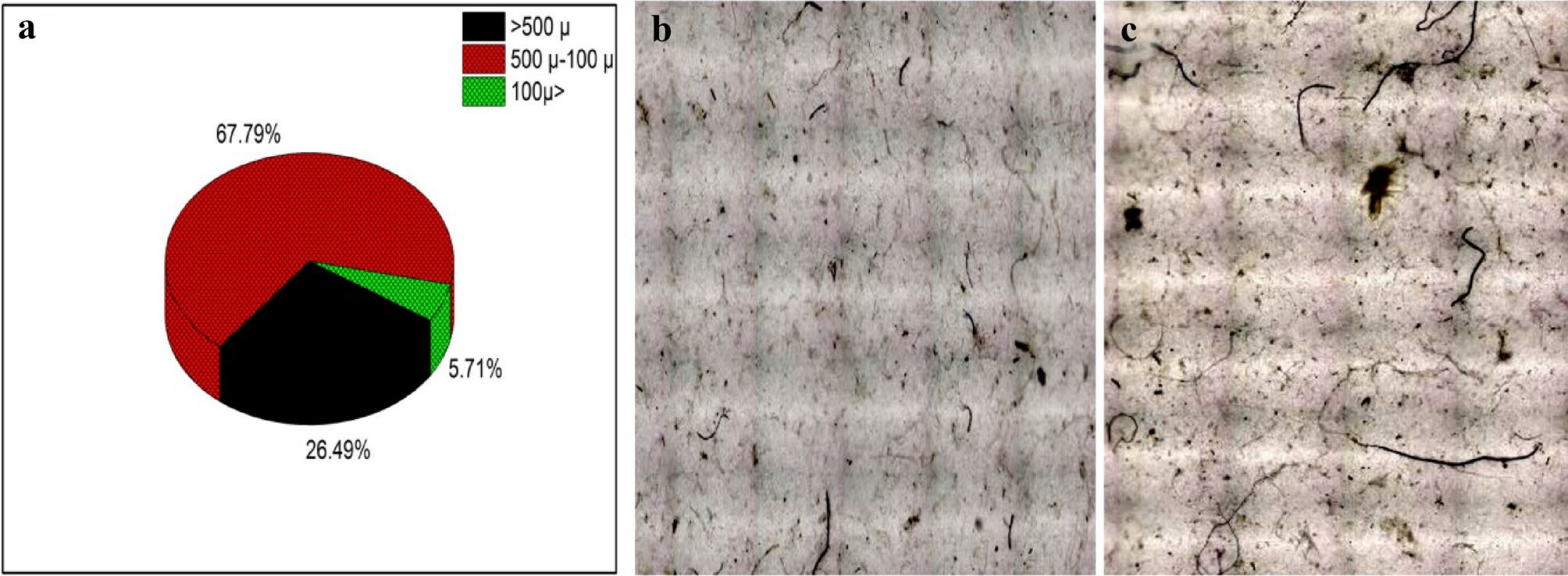
(**a**) size distribution of the microfibers extracted from the laundry wastewater. Optical microscopic images of the microfibers collected on membranes by (**b**) normal filtration method and (**c**) acetone assisted method.

Landry wastewater samples were subjected to the MP extraction procedure using acetone and were analysed using micro-FTIR spectroscopy. Acetone dissolved the complex organic contents of wastewater and leaves of the precipitates and microfibers. Smaller microfibers tend to float to the surface immediately after the addition of acetone whereas; the heavier and larger fibers remain on the bottom of the beaker. This makes the extraction process simpler and easier. Optical microscopic imaged of some of the extracted microfibers are presented in Fig. 6 and the size distribution of the microfibers is given in Fig. 7a. From the figures, it is evident that the microfibers extracted from the laundry wastewater are of varying sizes and colours. It was found that ~ 26.49% of the extracted microfibers was a size of above 500 µm, whereas 67.79% was a size between 500 and 100 µm and 5.71% was less than 100 µm. This shows that the size of microfibers generated in the laundry process are in various sizes.

Efficiency of acetone added method in the microfiber extraction from laundry wastewater was compared with the normal collection and filtration method by analysing the optical microscopic images (Fig. 7b,c). The microfiber collected on the membranes by using acetone in the process (Fig. 7c) was found to be cleaner than those samples prepared in the previous method (Fig. 7b). It can be attributed to that acetone dissolved the detergent residues and organic waste materials which were present in the laundry wastewater. As a result, the presences of interfering materials are limited, and the samples appear cleaner than normal. Cleaner samples devoid of interfering substances make the analysis and estimation of microfibers in the sample more accurate, easier and less biased.

### Sustainability of the extraction method

The present study demonstrated a novel simple and easier method of MPs extraction from face scrub and laundry wastewater. The process is less time consuming since acetone reacts with the samples quickly. Acetone is an organic solvent which belongs to the class 3 of guidelines established by International Council for Harmonization (ICH Q3C (R7) guidelines)^51^. According to the guideline, those organic solvents belong to class 3, and they have low toxic potential with no health hazards to human beings. Therefore, these solvents are good for the safety of the environment and human beings while the using for analytical purpose. The quantity of reagent used in the extraction process was very low such as 5 mL of acetone/sample. This method also does not require heating or membrane filtration of the samples. Thus, the energy and membrane material can be saved when comparing with the traditional methods of extraction of MPs from face scrub. In the case of microfiber extraction from laundry wastewater, it was found that the extracted microfibers from the samples were cleaner than traditional “collection and filtration method”. This will make the MP extraction and analysis easier and accurate and save the time required for the analysis.

The advantage of the proposed method is that it is lesser time (< 5 min), chemical consumption and it does not need any heating and filtration devices unlike the existing method. For instance, a previous method employed for the extraction of MPs from face cleansers utilised the digestion of the samples for a longer period of 5 days at 55 °C in 10% KOH solution^52^ with heating and filtration device. Moreover, the extraction efficiency of our proposed method in terms of recovery percentage could achieve highly at 94.1 ± 1.65%.

## Conclusion

A novel simple, less time consuming and easier method of MPs extraction from face scrub and laundry wastewater was demonstrated in this study. Thus, the derived method was made use to extract, quantify, analyse and characterise the MPs based on its type and size. The present study demonstrated a novel method of extraction with minimal reagents and set up to do the extraction of MPs more sustainable way. There are few countries initiated the ban of MPs usage in different products which are being used in daily life. Nevertheless, many countries are still not legally banned the usage. Hence, there is a need for simpler and easier extraction and analysis methods and a periodic assessment of MPs usage in all possible daily use products to limit their release to the environment and possible contamination.

## Data Availability

All data is included in this article. There is no supplemental data available in this article.
