# Author Correction: A novel simplified method for extraction of microplastic particles from face scrub and laundry wastewater

**DOI:** 10.1038/s41598-024-63484-z

**Published:** 2024-06-05

**Authors:** C. S. Shalumon, Chavalit Ratanatamskul

**Affiliations:** 1 Department of Environmental Engineering, Chulalongkorn University, Bangkok, Thailand; 2 Center of Excellence in Innovative Waste Treatment and Water Reuse, Faculty of Engineering, Chulalongkorn University, Bangkok, Thailand

Correction to: *Scientific Reports* 10.1038/s41598-023-41457-y, published online 29 August 2023

The original version of this Article contained an error, where 500 μm was incorrectly written as 500 nm.

As a result, in the Introduction,

“Generally, MPs in personal care products are in the form of “microbeads” which are plastic particles of less than 500 nm in diameter^24^.”

now reads:

“Generally, MPs in personal care products are in the form of “microbeads” which are plastic particles of less than 500 μm in diameter^24^.”

The original Article has been corrected.

